# Pesticide Residues in Commercial Lettuce, Onion, and Potato Samples From Bolivia—A Threat to Public Health?

**DOI:** 10.1177/1178630217704194

**Published:** 2017-04-18

**Authors:** Marlene Skovgaard, Susana Renjel Encinas, Olaf Chresten Jensen, Jens Hinge Andersen, Guido Condarco, Erik Jørs

**Affiliations:** 1 Fundación Plagbol, La Paz, Bolivia; 2 Dialogos, Copenhagen, Denmark; 3 Centre of Maritime Health and Society, University of Southern Denmark, Esbjerg, Denmark; 4 National Food Institute, Technical University of Denmark, Søborg, Denmark; 5 Department of Occupational and Environmental Medicine, Odense University Hospital, Odense, Denmark

**Keywords:** Environmental exposure/analysis, pesticide residues/analysis, vegetables/chemistry, consumer product safety, gas chromatography-mass spectrometry, Bolivia

## Abstract

Bolivia does not have a surveillance program for pesticide residues in food. The few published studies have suggested that pesticide contamination in food may present a public health problem. Data are lacking for all foods except tomatoes and breast milk. In this study 10 potato, 10 onion, and 10 lettuce samples from La Paz were sampled on August 15, 2015 at a local market and screened for 283 pesticides. Residues of cypermethrin, chlorpyrifos, difenoconazol, or/and λ-cyhalothrin were detected in 50% of the lettuce samples, whereas no pesticides were found in potatoes and onions. In 20% of the lettuce samples, the measurements were above the maximum residue limits, and 2 or 3 pesticides were identified simultaneously. Washing almost halved the pesticide levels, but still 20% of the samples showed measurements above the limits. No samples contained concentrations of pesticides which alone or together would lead to exposures that exceeded the acceptable daily intake or the acute reference dose. To protect consumers from pesticide poisonings and chronic effects, the development of measures for prevention, control, and monitoring of food contamination by pesticides in Bolivia is suggested.

## Introduction

Pesticides are chemical substances mainly used in agricultural pest control to improve yields and protect crops from being damaged.^1^ Due to a growing population and thus an increased need for food, pesticide import and use have been augmented in many countries, including Bolivia.^2^ This increase in combination with inadequate control with importers, a lack of education on safe pesticide handling,^3^ and how to expose of empty containers can cause severe adverse effects in pesticide users, consumers, and the environment.^1,3,4^

Pesticide intake in sufficient amounts can lead to acute poisonings and be so severe; it can cause coma and death.^5^ This is most often seen in connection with suicides or by occupational exposure,^3^ although it might happen by accidents among consumers as well.^5^ However, most of the adverse effects in consumers are caused by long-term intake of pesticide residues in food.^6^ These chronic effects include diminished IQ in individuals who were exposed to pesticides in utero^7^ and possibly diminished fertility.^8^ The cancer risk due to pesticides seems to be only slightly increased for consumers,^9^ and in any case, the risk caused by pesticide ingestion is lower than the benefit from eating fruits and vegetables.^10^ However, children, who generally have a greater food intake per body weight and thus a greater intake of pesticide residues per body weight, may have a greater cancer risk.^11,12^

Unlike the farmers and domestic pesticide spray workers who handle pesticides on a daily basis, many consumers are unaware of possible pesticide dangers.^13^ They depend on national food monitoring programs for their protection from dietary contamination. However, no national surveillance program exists in Bolivia where the level of pesticide residues in agricultural products is largely unknown.

Through memberships of Food and Agriculture Organization of the United Nations (FAO) and World Health Organization ( WHO) and according to own laws (Nueva Constitución Política del Estado),^14^ Bolivia should comply with the crop-specific maximum residue limits (MRLs) stated by the Codex Alimentarius Commission (Codex). Furthermore, pesticide short-term intake ought to not exceed the acute reference doses (ARfDs), and pesticide long-term intake should be below the acceptable daily intake (ADI) limits recommended by the FAO/WHO Joint Meetings on Pesticide Residues (JMPR) to avoid negative health effects.^15–17^

Although hundreds of studies and national pesticide residue surveillance programs have been conducted and published worldwide, especially in the high-income countries,^18–22^ only 3 pesticide residue studies from Bolivia have been published previously: 1 in breast milk^23^ and 2 in tomatoes.^24,25^ Data from all other food products are missing. The studies in Bolivian tomatoes conducted by FAO^24^ and Plagbol^25^ demonstrate that more than 50% of the samples contained pesticide residues. A weakness in these studies was that they were conducted in Bolivian laboratories which do not have long-term experience with pesticide residue analysis, were not able to screen for multiple pesticides, and did not have international accreditation.

We conducted this study in lettuce, onion, and potatoes to explore possible pesticide residues in other vegetables than tomatoes. Onion (*Allium cepa, ruber*), potato (*Solanum tuberosum* subsp *andigena*) and lettuce (*Lactuca sativa* var *longifolia*) were selected for the following reasons: All Bolivians, independent of social status, eat these vegetables year round, and in the case of lettuce and onion, they are frequently consumed raw. Furthermore, tuber vegetables, of which potatoes constitute an important part, are major contributors to the diet in Bolivia.^26^

With the former studies^24,25^ in mind, we hypothesized that the MRLs, the ADIs, and the ARfDs were surpassed—alone and in combination in at least some samples. We analyzed both crude samples and samples prepared for consumption to get a closer estimate of the actual dietary health risk and compared the results with other lower middle-income countries as well as to other South American countries.

## Methods

### Settings

This study on pesticide residues in vegetables was conducted during August of 2015 in La Paz. Most of the vegetables consumed in La Paz arrive at the central market, named Rodríguez, several times per week. They are transported in shared trucks from different rural Bolivian communities: from the tropical and humid east 400 meters above sea level (m a.s.l.) to the dry highlands in the west more than 4000 m a.s.l. Furthermore, products arrive from Chile, Peru, and other countries nearby. From the Rodríguez market, the vegetables are distributed to the smaller markets and to the supermarkets all over town.

### Vegetable consumption and preparation data

In May/June of 2015 a survey (see Supplementary Appendix 1) was applied to 55 vegetable consumers and 36 vegetable and fruit vendors to find out how the La Paz inhabitants usually prepare their vegetables and to calculate the estimated daily intake of lettuce, onion, and potato. The WHO and FAO do not have official consumption data from Bolivia.^27,28^ Included consumers and vendors were those who showed up on a neighborhood or a vendor meeting, respectively, after oral invitation. The neighborhood was chosen randomly by a drawing out of 5 medium-sized neighborhoods in La Paz, and the vendors all worked at a smaller La Paz market chosen by our local community partner Control Social. Median age of respondents was 50 (interquartile range: 39-59) years. Child consumption data were provided by adult respondents on the basis of children in their household. The questionnaire was made up of a short background section (age, sex, and occupation) and multiple choice questions, written in Spanish and autocompleted by respondents. A pilot test was conducted on 10 people for uniform understanding, and 2 authors were present at the meetings to clarify eventual doubts. The vegetable preparation procedure which is indicated by most of the responders is shown in Table 1.

**Table 1.** Preparation of vegetable samples bought in La Paz, Bolivia, 2015.

	Potato	Lettuce	Onion
Preparation according to MRL compliance	Whole and raw but without soil (they were gently washed in running water or cleaned with a brush)	Whole but without damaged leaves, soil, or roots	*Dry*: whole but without the dry shell, soil, or roots *Fresh*: whole but without roots and soil
Preparation for consumption^a^	Peeled, washed, and boiled 30 min in 3600 m a.s.l. (73%)	Outer leaves removed. Then washed in a bowl of water for 5 min (66%)	The most external onion layers removed. Then washed in a bowl of water for 5 min (31%)

Abbreviations: m a.s.l., meters above sea level; MRL, maximum residue limit.

Adapted from European Union Directive 90/642^29^ and own survey.

a The percentage of responders preparing the vegetables using the mentioned procedure is shown in parenthesis.

### Vegetable sampling

All vegetables were bought Saturday morning on August 15, 2015. Saturday was chosen because it is the time of the week where most vegetables are fresh and because most of the La Paz inhabitants go to the market during the weekend. August was chosen because it was the month that best fitted with the remaining project activities planned by the authors. As August is part of the dry season/winter in Bolivia, it is not the season where most pesticides are used.

Each type of vegetable was bought from 2 distinct supermarket chains and 8 different vendors in the Rodríguez market. That is, 10 primary samples per vegetable type. As each vendor did not sell all 3 types of vegetables, samples were obtained from a total of 24 vendors. Approximately, 60 to 120 vendors or re-vendors sell each of the 3 vegetables, however, only representing 8 to 12 distinct geographic regions per vegetable.

From each vendor, 1 primary sample of 2.5 kg of potatoes, 20 to 25 onions, or 10 lettuce heads were purchased. The vendors and supermarkets were not informed that we were going to show up on the given day. Each sampled lot was randomly chosen without any specific selection criteria while ensuring that they originated from different Bolivian and Peruvian geographic regions and were as fresh as possible. Vendors chose and packed the vegetables with their bare hands in their own plastic bags. Afterward, the sample was placed in another clean, large, and appropriately numbered bag to ensure proper identification and separation of samples. Until preparation, vegetables were stored at room temperature (15-20°C).

### Vegetable preparation

Samples were prepared 2 days after buying the vegetables because many La Paz inhabitants store vegetables several days before use. The content of each vegetable bag was split into 2 subsamples with at least 1 or 2 kg and at least 10 or 5 units according to the Codex.^30^ One sample was prepared to eat, and the other sample was left almost untouched, removing only the obviously inedible parts (see Table 1).^29^ Thus, we ended up with 60 vegetable subsamples.

After preparation, the vegetables were minced in a food processor. Then, 150 g of the blended vegetable mass was put in a numbered container, previously sterilized in boiling water, and frozen before airplane transport to Lima the following day. Samples were accompanied by one of the authors and kept frozen in a polystyrene box with gel packs on the journey. Due to it being illegal to transport whole, fresh vegetables from Bolivia to Peru, the preparation and laboratory procedures were divided.

### Laboratory analysis

The Andes Control laboratory in Lima, Peru (which has later changed name to Inspectorate Services Perú S.A.C.), was chosen because of its closeness to La Paz, its international accreditation (DAkkS Germany: ISO 17.025),^31^ and its more than 10 years of experience in pesticide analysis in food including vegetables. Samples were analyzed by a multiresidue procedure using gas chromatography with mass spectrometry (GC/MS) and QuEChERS (Quick, Easy, Cheap, Effective, Rugged, and Safe) method.^32^ This analysis was chosen because it included many of the pesticides reported to be used in Bolivia in general^3,25,33^ and for lettuce, onion, and potatoes specifically (please see Supplementary Appendix 2). During 3 days, lettuce, then potato, and finally onion samples were analyzed. Analyses of 283 active pesticide ingredients from all major chemical pesticide classes (benzimidazoles, carbamates, dicarboximides, organochlorines, organophosphates, pyrethroids, triazoles, etc.) with a detection limit for almost all substances of 0.003 mg/kg and a quantification limit of 0.01 mg/kg of product was conducted (see Supplementary Appendix 3). The recovery for all substances was between 70% and 120%. Because results in this article are used for risk assessment rather than law enforcement, the actual measured values as reported from the laboratory have been used, as recommended by the SANTE guidelines.^34^ That is, the uncertainty factor has not been taken into account.

### Outcomes and statistics

The primary outcomes were concentration levels and types of pesticides detected compared with the pesticide-specific and crop-specific MRLs mentioned on the Web page for the Codex.^16^ Where the Codex did not mention the crop-specific MRL, limits from the United States and the European Union were consulted^35,36^ (Table 2). Secondary outcomes were acute and chronic hazard quotients (HQs) calculated as the ratio between the estimated daily intake of a pesticide per kilogram body weight and the ARfD and the ADI, respectively. Calculations were made for children and adults separately (Table 3):

$${\mathrm{HQ}}_{\mathrm{acute}}=\frac{\mathrm{Estimated daily intake}\left(\mathrm{100th}\;\mathrm{percentile}\right)}{\mathrm{ARfD}}$$

and

$${\mathrm{HQ}}_{\mathrm{chronic}}=\frac{\mathrm{Estimated daily intake}\left(\mathrm{50th}\;\mathrm{percentile}\right)}{\mathrm{ADI}}$$

**Table 2.** Pesticide residues (mg/kg) detected per lettuce sample bought in La Paz, Bolivia, 2015, in relation to the MRLs stated by CAC, EU, and United States (*n* = 10^a^).

	MRL			Lettuce 3		Lettuce 4		Lettuce 6		Lettuce 7		Lettuce 9	
Pesticide^b^	CAC^16^	EU^35^	USA^36^	C	W	C	W	C	W	C	W	C	W
Cypermethrin	0.7	—	—	0.21	0.10	1.36	0.79	ND	ND	ND	ND	ND	ND
Chlorpyrifos	—	0.05	0.1	0.24*	0.21*	0.73*	0.30*	ND	ND	ND	ND	ND	ND
Difenoconazole	2.0	2.0	—	0.44	0.29	ND	ND	ND	ND	ND	ND	ND	ND
λ-cyhalothrin	—	0.5	2.0	ND	ND	ND	ND	0.03	ND	0.17	0.10	0.39	0.19

Abbreviations: C, crude; CAC, Codex Alimentarius; EU, European Union; MRL, maximum residue limit; ND, not detected; W, washed.

a Only samples with detected pesticides are shown in the table.

b Only pesticides detected are listed. For the full list of the 283 pesticides, please see Supplementary Appendix 3.

* Significantly exceeds MRLs (relative expanded uncertainty of 50% assumed).^34^ Residue measurements are not corrected for uncertainty or bias.

**Table 3.** Short-term and long-term pesticide intake for adults and children in La Paz, Bolivia, compared with CAC’s acute reference dose and acceptable daily intake.

	Cypermethrin^37^	Chlorpyrifos^38^	Difenoconazole^39^	λ-cyhalothrin^40^
Estimated daily intake, 100th percentile, in relation to the acute reference dose (% HQ_acute_)				
Ref.	0.04 mg/kg bw	0.1 mg/kg bw	0.1 mg/kg bw	0.02 mg/kg bw
Adult	0.003 mg/kg bw (7.4%)	0.002 mg/kg bw (1.6%)	0.001 mg/kg bw (1.0%)	0.001 mg/kg bw (4.3%)
Child	0.008 mg/kg bw (20.4%)	0.004 mg/kg bw (4.4%)	0.003 mg/kg bw (2.6%)	0.002 mg/kg bw (11.7%)
Estimated daily intake, 50th percentile, in relation to the acceptable daily intake (% HQ_chronic_)				
Ref.	<0.02 mg/kg bw	<0.01 mg/kg bw	<0.01 mg/kg bw	<0.02 mg/kg bw
Adult	0.00007 mg/kg bw (0.4%)	0.00004 mg/kg bw (0.4%)	0.00002 mg/kg bw (0.2%)	0.00002 mg/kg bw (0.1%)
Child	0.0004 mg/kg bw (2.0%)	0.0002 mg/kg bw (2.2%)	0.0001 mg/kg bw (1.3%)	0.0001 mg/kg bw (0.6%)

Abbreviations: ADI, acceptable daily intake; ARfD, acute reference dose; bw, body weight; CAC, Codex Alimentarius.

Besides calculating the average pesticide short-term intake of the whole sample which consists of 5 to 10 vegetable units,^30^ we also calculated the theoretically extreme values for 1 unit of vegetables multiplying by a variability factor of 3 recommended by FAO.^11^ In multicontaminated samples, the hazard index (HI) was calculated to express a cumulative intake without accounting for if 2 pesticides belonged to the same group or had the same mode of action:

$$\mathrm{HI}=\sum_{i}^{n}{\mathrm{HQ}}_{i}$$

Hazard index lower than 1 (=lower than 100%) indicated no apparent human harm. The data were tabulated in and analyzed with STATA Software (version 11.1; StataCorp, College Station, TX, USA) and Microsoft Excel (version 2010).

Estimated daily intake, 100th percentile, is the maximum exposure of pesticides in 1 day for the most lettuce-eating individuals, whereas estimated daily intake, 50th percentile, is the average exposure of pesticides per day. These results are based in *most* contaminated samples. The results do not include the variability factor.

The study was reviewed by the Bolivian public institutions SENASAG, INLASA, and Control Social prior to initiation.

## Results

### Pesticide residue concentrations

**Figure 1.**
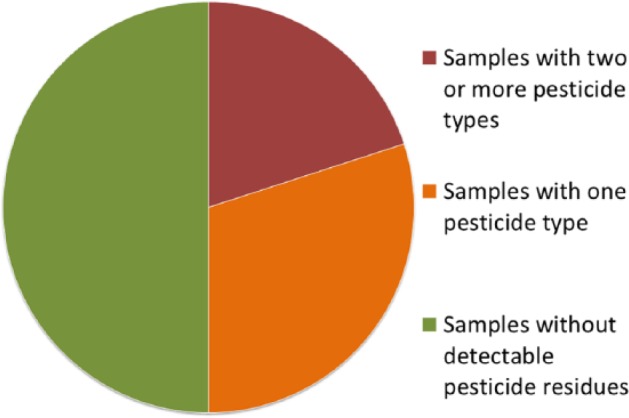
Number of pesticide residues identified in unwashed lettuce samples (*n* = 10) bought in La Paz, Bolivia, 2015.

No pesticide residues were found in potato or onion samples. In lettuce, pesticide residues were detected in 5 out of 10 unwashed samples (Figure 1). After washing, it went down to 4 out of 10. “Lettuce 3” contained cypermethrin, chlorpyrifos, and difenoconazole; “Lettuce 4” had cypermethrin and chlorpyrifos; and “Lettuce 6, 7, and 9” contained λ-cyhalothrin (Table 2). It should be noted that none of the pesticides identified belonged to the most hazardous pesticide groups, WHO Class 1a or 1b^41^ and that of the positive samples 5/5 contained a pyrethroid and 2/5 an organophosphate.

The relationship between the level of pesticides identified in each lettuce sample and the lettuce-specific MRLs is shown in Table 2. Regardless of the type of pesticide, the pesticide concentration dropped approximately 45% (minimum 13%, maximum 70%) after washing lettuce for 5 minutes in a water bath. Still, the pesticide concentration in “Lettuce 3 and 4” showed measurements above the MRLs in both washed and unwashed samples. Although both potato and onion samples included a sample from Peru, all lettuce samples originated from Bolivia, maximum 2 hours’ drive from the city of La Paz. Among the contaminated samples, 1 was bought in a supermarket, 2 were grown in tents, and 2 were grown in the Altiplano. The most contaminated samples were grown in warmer areas below 3000 m a.s.l.

### Human health risk calculations

According to our survey, the average estimated daily intake of lettuce was 3 g per person per day for children and adults, whereas the large portion was 60 and 120 g. Assuming an adult weight of 55 kg and a child weight of 10 kg, the 50th percentile estimated daily intake per kilogram body weight was 0.055 and 0.4 g for adults and children, respectively. The 100th percentile estimated daily intake of lettuce per kilogram body weight was 2.18 and 6.0 g for adults and children.

Table 3 shows what pesticide quantities these people would have eaten per kilogram body weight, had they eaten the most contaminated lettuce, and correlate these findings with the ARfDs and ADIs. Highest pesticide value was chosen instead of average content for both ARfD and ADI calculations to give the most conservative estimates. Even so, neither the HQ_acute_ nor HQ_chronic_ are greater than 100%; that is, the chronic and acute exposures do not exceed ADIs or ARfDs, respectively, even if HQ_acute_ is multiplied by the variability factor of 3.^11^

Worst-case scenario HI results calculated for the most contaminated samples show that HI_acute_ is 2.6% and 9.0% for the 100th percentile adult had “Lettuce 3” and “Lettuce 4” been eaten, respectively, whereas HI_chronic_ is 0.4% and 0.8%. For the 100th percentile child, HI_acute_ is 7.2% and 25% for “Lettuce 3” and “Lettuce 4,” respectively, whereas HI_chronic_ was 2.4% and 4.2%. Thus, these results show that the HI for the acute and chronic exposure is below 100% for all lettuce samples.

## Discussion

The results of this study do not demonstrate an unacceptable health problem for Bolivian consumers when ingesting lettuce, onions, and potatoes bought in La Paz but show that 20% of washed and nonwashed lettuce samples contain pesticide levels above the MRLs.

The MRL exceedance rate found in our lettuce samples is less than the approximately 60% contaminated samples found in a PLAGBOL study from 2012^25^ in Bolivian tomatoes bought directly from farmers in Cochabamba and Chuquisaca and about the same as found by FAO/INLASA^24^ in tomatoes bought in La Paz city. However, a rate of 20% is still concerning, and higher than levels found in lettuce commercial lettuce samples produced in high-income countries.^18–21,42^ Similar to Bolivia, pesticide residue surveillance studies from other South American countries (except Brazil) and other low-income and lower middle-income countries^43^ worldwide are sparse. A PubMed and Google search on pesticide residues in lettuce from low-income and lower middle-income countries only identified a total of 3 studies. Two studies from Ghana^44,45^ and 1 study from Senegal^46^ where 55% to 84% and 27% of lettuce samples, respectively, showed measurements above the MRLs, but none were above the ADIs.^44^ Lettuce studies from other South American countries, which all belong to the groups of high-income or higher middle-income countries, found MRL exceedance rates of 1% to 13%,^18,19,42,47^ whereas the ADI was not surpassed^18^ and 6% of samples were above the ARfD.^18^ Although it should be remembered that these studies vary widely in methodology, pesticides included, and number of lettuce samples analyzed, there is a clear tendency toward higher MRL exceedance rates in lower income countries. The ARfD and ADI compliances were only investigated in a few studies.^18,44^ It is well known that MRLs differ between countries/institutions, years, and crops.^16,35,36^ Despite these alterations, the MRLs continue to be a more uniform tool for comparison between studies than ARfD, ADI, and HI, as MRL only depends on the pesticide concentration and current laws, whereas the other measures are related to consumption patterns and body weight, that is, cultural, sociological, economic, geographic, and individual factors.

In our study, using consumption data based on our survey in La Paz, worst-case estimates of exposure from pesticide residues in lettuce did not exceed the ADI or ARfD for individual pesticides. Conservative estimates of cumulative exposure using the HI method on the 2 multicontaminated lettuce samples from our study did show HI values up to 25% for children, which, though rather high, do not indicate an unacceptable risk for the consumers (Table 2). To cause a health risk when eating the most contaminated lettuce sample, “Lettuce 4,” the 50th and 100th percentiles of estimated daily lettuce intake would have to be 7 g/kg body weight every day and 24 g/kg body weight on a single occasion, respectively. These calculations do not account for pesticide content in other parts of the diet, and do not also include a variability or uncertainty factor,^34,48^ but are conservative because they are based on the single most contaminated sample, do not include reduction in pesticide concentration due to washing, and sum up all pesticides without considering chemical class. Furthermore, a daily average lettuce intake of 7 g/kg body weight and a large portion of 24 g/kg body weight are very far from the results of our consumption questionnaire and quite far from the consumption average and extreme of most people, including neighboring Brazil, according to WHO/FAO.^27,28^ Nonetheless, children from the Netherlands, the Chinese general population, and elderly people from Hungary would be at risk due to their specific eating patterns.^27,28^ Thus, although ADIs and ARfDs are not surpassed, MRL breaches are absolutely not acceptable due to the differences in dietary habits between individuals and accumulated pesticide exposure from the whole diet.

Because of the increased pest vulnerability of lettuce compared with potato and onion, and due to lettuce not being suitable for long-term storage, it is not surprising that we find more pesticides on lettuce. However, we had feared to detect organochlorines in all 3 crops due to lack of farmer education and poor habits. Fortunately, we did not find pesticides from this group or any other obsolete pesticides as was the case in other Bolivian studies,^25,33^ but clearly we cannot exclude that they were present below the detection limits. Former Bolivian studies have reported that pyrethroids and organophosphates are the 2 most common groups of pesticides used in the agriculture.^3,33^ That is coherent with our findings and lettuce results from Ghana^44,45^ and Senegal,^46^ but not to the results from Brazil,^47^ Canada,^21^ and Denmark.^20^ Half of these studies also found quite a high prevalence of organochlorines.^21,44,46^ Both organophosphates and pyrethroids are metabolized relatively fast in the human body to nontoxic components, for which reason they are primarily known for their acute toxicity.^49,50^

This study shows that washing the lettuce 5 minutes in a water bath lowered the pesticide concentration by almost 50%. Similar results have been shown in tomatoes when washing and even more so after peeling.^25^ This is also in concordance with review studies on different food preparation methods.^51,52^ Thus, until a national pesticide residue monitoring program is in place in Bolivia, consumers will have to rely fully on their own initiatives, such as washing and peeling, for food protection. From personal encounters with the Bolivian governmental entity SENASAG, we know that they plan the implementation of a pesticide monitoring program within the next years. However, it is important to assure that a future program will include and prioritize not only export but also domestic products, as pesticide residue monitoring programs from Bolivia’s neighboring countries tend to separate the 2 groups of products^18,19,47,53,54^ and place most emphasis on export products.^53^

### Strengths and weaknesses

This is the first Bolivian study to show results of pesticide residues in 3 types of vegetables and it is also one of the few existing pesticide residue studies done in Bolivia so far. The strengths of this study include the sample size; the representativeness of almost all geographic areas which produce and sell potatoes, onions, and lettuce in La Paz; and the design, which is similar to the shopping and preparation habits of the inhabitants. Furthermore, the analyses themselves screened for 283 different active pesticide ingredients and were conducted in an internationally accredited laboratory with more than 10 years’ experience.

Limitations on the estimates of residue concentrations and types include that all samples were obtained on one single day during a whole year instead of various days, a division of the preparation/laboratory process in 2 parts, and storage at room temperature between obtaining and processing the samples. Samples were analyzed on 3 separate days instead of 1 but were frozen on the same day which should diminish the significance of the possible error.^55^ Gas chromatography with mass spectrometry was the only method used. It identifies almost all relevant pesticides according to our experience^3,25,33^ but is naturally not exhaustive. No double detection was used, and information on possible matrix effect^22^ was not available from the laboratory. However, the laboratory was accredited to international standards. In addition, no precise time span between harvest of the vegetables and arrival on the market/supermarket was known. This may have affected especially the findings in onions and potatoes as they might have been stored for month, although they had arrived on the market recently. All the mentioned limitations may have caused bias in residue concentration and/or number of residue types; however, the exact magnitude is hard to estimate.

## Conclusions

Despite the limitations mentioned, our investigation shows that half of the lettuce samples bought in La Paz, Bolivia, contained 1 or more pesticides, and one-fifth were above the MRLs. None of the lettuce samples contained concentrations of pesticides which led to exposures that would exceed the ADI or ARfD alone or in combination. Nevertheless, it should be remembered that an individual’s accumulated pesticide exposure is the sum of pesticide residues in the entire diet and not only in lettuce. In onion and potatoes, no pesticides were detected. However, this may be due to one or more limitations as, for instance, taking the samples during the dry season.

Although the results for onions and potatoes are comforting, and better than hypothesized, the frequency of measured residues above the MRL in the lettuce samples is higher than in high-income and neighboring countries and underlines an existing problem: lack of education, communication, and control of pesticide management in Bolivian agriculture. Measures should be taken to improve the food safety for consumers in La Paz, Bolivia, and ensure that standards are met. This includes working on the endorsement and accreditation of Bolivian laboratories, conducting further pesticide residue investigations and implementing a national pesticide monitoring program.

## Supplementary Material

Supplementary material
