# Supplementary material for: Pesticide Residues in Commercial Lettuce, Onion, and Potato Samples From Bolivia—A Threat to Public Health?
Source: Environ Health Insights. 2017 Apr 18;11:1178630217704194. doi: 10.1177/1178630217704194 (PMC5400016; doi:10.1177/1178630217704194)
Supplement: Supplementary material [file Supplementary_Data_704194.pdf]

Supplementary data

Appendix 1: Survey on vegetable consumption and preparation applied to 55 vegetable consumers and 36 vegetable and fruit vendors in La Paz, Bolivia, 2015.

a. How old are you? ____________ years

b. What is your sex? Female ☐ Male ☐

c. Thinking about the last month, what do you or your family members do generally when you have bought vegetables? (Marc with an X – **only one time**)

We use them immediately	We store them

d. Thinking about the last month, how do you or your household members generally prepare the following vegetables before eating them? (Marc with an X in **every row**)

Vegetable	I eat them as they are	I wash, peal and boil them	I wash and boil them	I wash and peal them	I boil them	I peal them	I wash them
Lettuce							
Tomato							
Potatoes							
Onion							
Carrots							

e. Thinking about the last month, how many times per week do you generally eat the following vegetables? (Marc with an X in **every row**)

Vegetable	(Almost) every day of the week	3 to 6 times per week	1 to 2 times per week	Less than once a week	Never
Lettuce					
Tomato					
Potatoes					
Onion					
Carrots					

f. What amount of the following vegetables do **you** eat **in one single day** as maximum? (Marc with an X in **every row**)

Vegetable	I never eat it	A little bit	¼ plate	½ plate	1 plate
Lettuce					
Tomato					
Potatoes					
Onion					
Carrots					

g. How much do you weigh? (Marc with an X – **only one time**)

Less than 50 kilograms	51 to 60 kilograms	61 to 70 kilograms	More than 70 kilograms

h. If THERE ARE NO children in your household below the age of 12 years, skip the rest of the questions".
If THERE IS a child in your household below the age of 12 years, please answer the following questions. If there is more than one child, answer the questions thinking about

the child that weighs closest to 15 kilograms and that is younger than 6 years old: What amount of the following vegetables does **this child** eat **in one single day** as maximum? (Marc with an X in **every row**)

Vegetable	He/she never eats it	A little bit	¼ plate	½ plate	1 plate
Lettuce					
Tomato					
Potatoes					
Onion					
Carrots					

i. How old is the child?______ years.

j. How much do you think the child weighs? (Marc with an X – **only one time**)

Less than 10 kilograms	11 to 20 kilograms	21 to 30 kilograms	31 to 40 kilograms	More than 40 kilograms

Appendix 2: Pesticides used for potato, onion and lettuce cultivation in Rio Abajo, near La Paz

Potato		***Onion***		***Lettuce***	
Aldrin*	1 time, 1kg per 50kg of potatoes	Metalaxyl-M*	2-3 times, 12 tablespoons	Metalaxyl-M*	2 times, 5 tablespoons
Parathion methyl*	2 times, 160 mL per liter	Mancozeb	2-3 times, 12 tablespoons	Mancozeb	2 times, 5 tablespoons
Aldicarb	1 time, 2-3 tablespoons	Profenofos*	1 time, 5 tablespoons	Profenofos*	2 times, 10 tablespoons
Cartap	1 time, 2 tablespoons	Chlorothalonil*	1 time, 1 tablespoons	Methamidophos*	2 times, 6 tablespoons
Methamidophos*	3 times, 3-6 tablespoons				
Cypermethrin*	2 times, 1 lid and 3 tablespoons				
Difenoconazole*	1 time, 5 tablespoons				

Source: Data supplied by the Plagbol agronomist, 2009/2010 (unpublished data).
Where the dose is mentioned in tablespoons, it is per 20 Liters of water, equal to the content of a spray man's backpack.
*Pesticides included in the 283 multi-residue analysis applied in the present study.

Appendix 3: The 283 active pesticide substances screened for in the study using QuEChERs method and GC with MS in the laboratory Andes Control, Peru. For each substance the LOD and LOQ are mentioned in mg per kg of matrix.

Pesticide	LOD	LOQ	Pesticide	LOD	LOQ	Pesticide	LOD	LOQ	Pesticide	LOD	LOQ
2-Phenylphenol	0.003	0.01	DDE-p,p’	0.003	0.01	Flusilazole	0.003	0.01	Piperonyl butoxide	0.003	0.01
2,3,5,7-Tetrachlorphenol	0.003	0.01	DDT-o,p’	0.003	0.01	Flutolanil	0.003	0.01	Pirimicarb	0.003	0.01
3,4-dichloro aniline	0.003	0.01	DDT-p,p’	0.003	0.01	Flutriafol	0.003	0.01	Pirimiphos ethyl	0.003	0.01
Acephate	0.006	0.02	Decabromodiphenyl ether	0.003	0.01	Folpet	0.003	0.01	Pirimiphos methyl	0.003	0.01
Aclonifen	0.006	0.02	Deltamethrin	0.003	0.01	Formothion	0.003	0.01	Prochloraz	0.003	0.01
Acrinathrin	0.003	0.01	Demeton (O+S)	0.003	0.01	Furalaxil	0.003	0.01	Procymidone	0.003	0.01
Alachlor	0.003	0.01	Desethylatrazin	0.003	0.01	Furmecyclox	0.003	0.01	Profenofos	0.003	0.01
Aldrin	0.003	0.01	Desetilterbutilazin	0.003	0.01	Haloxyfop-p-methyl	0.003	0.01	Prometrine	0.003	0.01
Allethrin	0.003	0.01	Desmedipham	0.006	0.02	HCB (Hexachloro-benzene)	0.003	0.01	Propachlor	0.003	0.01
Amitrole	0.003	0.01	Desmetryne	0.003	0.01	HCH-alfa	0.003	0.01	Propamocarb	0.003	0.01
Atrazine	0.003	0.01	Diazinon	0.003	0.01	HCH-beta	0.003	0.01	Propargite	0.003	0.01
Atrazine-desisopropyl	0.003	0.01	Dichlobenil	0.003	0.01	HCH-delta	0.003	0.01	Propazine	0.003	0.01
Azaconazole	0.003	0.01	Dichlofluanid	0.003	0.01	Heptachlor	0.003	0.01	Propiconazole	0.003	0.01
Azinphos-ethyl	0.003	0.01	Dichloprop	0.003	0.01	Heptachlor epoxide-endo	0.003	0.01	Propoxur	0.003	0.01
Azinphos-methyl	0.003	0.01	Dichloran	0.003	0.01	Heptachlor epoxide-exo	0.003	0.01	Propyzamide	0.006	0.02
Azoxystrobin	0.003	0.01	Dichlorobenzamide	0.003	0.01	Heptenophos	0.003	0.01	Proquinazid	0.003	0.01
Benalaxyl	0.003	0.01	Dichlorvos	0.003	0.01	Hexabromcyclodo-decan	0.003	0.01	Prosulfocarb	0.003	0.01
Benfuracarb	0.003	0.01	Diclobutrazol	0.003	0.01	Hexaconazole	0.003	0.01	Prothiofos	0.003	0.01
Bentazone	0.003	0.01	Dicofol	0.003	0.01	Hexithiazox	0.003	0.01	Pyrazophos	0.003	0.01
Bifenthrin	0.003	0.01	Dieldrin	0.003	0.01	Imazalil	0.003	0.01	Pyrethrins (mixed)	0.003	0.01
Bioallethrin	0.003	0.01	Diethofencarb	0.003	0.01	Indoxacarb	0.003	0.01	Pyridaphenthion	0.006	0.03
Biphenyl	0.003	0.01	Difenoconazole	0.003	0.01	IPBC	0.003	0.01	Pyrifenox	0.003	0.01
Bitertanol	0.003	0.01	Diflubenzuron	0.003	0.01	Iprobenphos	0.003	0.01	Pyrimethanil	0.003	0.01
Bromacil	0.003	0.01	Diflufenican	0.003	0.01	Iprodione	0.003	0.01	Pyriproxifen	0.003	0.01
Bromophos-ethyl	0.003	0.01	Dimethenamid	0.003	0.01	Kresoxim-methyl	0.003	0.01	Quinalphos	0.003	0.01
Bromophos-methyl	0.003	0.01	Dimethoate	0.003	0.01	Lenacil	0.003	0.01	Quinometionate	0.003	0.01
Bromopropylate	0.003	0.01	Dimethomorph	0.003	0.01	Lindane	0.003	0.01	Quinoxyfen	0.003	0.01
Bromoxynil	0.003	0.01	Diniconazole	0.003	0.01	Linuron	0.006	0.02	Quintozene	0.003	0.01
Bupirimate	0.003	0.01	Dinoseb	0.003	0.01	Lufenuron	0.006	0.02	Quizalofop-ethyl	0.006	0.03
Buprofezin	0.003	0.01	Diphenylamine	0.003	0.01	Malathion	0.003	0.01	S421	0.003	0.01
Cadusafos	0.003	0.01	Disulfoton	0.003	0.01	Mecarbam	0.003	0.01	Sebuthylazine	0.003	0.01
Captan	0.003	0.01	Diuron	0.006	0.02	Mepanipyrim	0.003	0.01	Silafluofen	0.003	0.01
Carbaryl	0.003	0.01	Endosulfan-alfa	0.003	0.01	Metalaxyl	0.003	0.01	Simazine	0.003	0.01
Carbofuran	0.003	0.01	Endosulfan-beta	0.003	0.01	Metamitron	0.003	0.01	Spiromesifen	0.003	0.01
Carbon tetrachloride	0.003	0.01	Endosulfan-sulphate	0.003	0.01	Metazachlor	0.003	0.01	Spiroxamine	0.003	0.01

Carbophenothion	0.003	0.01	Endrin	0.003	0.01	Methamidophos	0.006	0.02	Sulfotep	0.003	0.01
Carbosulfan	0.003	0.01	EPN	0.003	0.01	Methidathion	0.003	0.01	Sulfur (S8)	0.03	0.10
Carfentrazone-ethyl	0.003	0.01	Epoxiconazole	0.003	0.01	Methiocarb	0.003	0.01	Tau-Fluvalinate	0.003	0.01
Chlordane, alfa	0.003	0.01	Esfenvalerate	0.003	0.01	Methoxychlor	0.003	0.01	Tebuconazole	0.003	0.01
Chlordane, gama	0.003	0.01	Ethion	0.003	0.01	Metolachlor	0.003	0.01	Tebufenpyrad	0.003	0.01
Chlorfenapyr	0.003	0.01	Ethofumesate	0.003	0.01	Metrafenone	0.003	0.01	Tebutam	0.003	0.01
Chlorfenson	0.003	0.01	Ethoprophos	0.003	0.01	Metribuzin	0.003	0.01	Tecnazene	0.003	0.01
Chlorfenvinphos	0.003	0.01	Etofenprox	0.003	0.01	Mevinphos	0.003	0.01	Teflubenzuron	0.003	0.01
Chloridazon	0.003	0.01	Etridiazole	0.003	0.01	Monocrotophos	0.003	0.01	Tefluthrin	0.003	0.01
Chlorobenzilate	0.003	0.01	Etrimfos	0.003	0.01	Moschus-Keton	0.003	0.01	Terbacil	0.003	0.01
Chlorocresol	0.003	0.01	Famoxadone	0.003	0.01	Myclobutanil	0.003	0.01	Terbufos	0.003	0.01
Chloronaph-thalenes	0.003	0.01	Fenamiphos	0.003	0.01	Naled	0.003	0.01	Terbuthylazine	0.003	0.01
Chlorothalonil	0.003	0.01	Fenarimol	0.003	0.01	Napropamide	0.003	0.01	Terbutryn	0.003	0.01
Chlorpropham	0.003	0.01	Fenazaquin	0.003	0.01	Nitrofen	0.006	0.02	Tetrachloroanisole	0.003	0.01
Chlorpyrifos	0.003	0.01	Fenbuconazole	0.003	0.01	Nuarimol	0.003	0.01	Tetraconazole	0.003	0.01
Chlorpyrifos methyl	0.003	0.01	Fenchlorphos	0.003	0.01	Omethoate	0.003	0.01	Tetradifon	0.003	0.01
Chlorthion	0.003	0.01	Fenhexamid	0.003	0.01	Oxadiazon	0.003	0.01	Tetramethrin	0.003	0.01
Chlortoluron	0.003	0.01	Fenitrothion	0.003	0.01	Oxadixyl	0.003	0.01	Thiabendazole	0.003	0.01
Chlozolinate	0.003	0.01	Fenoxaprop - ethyl	0.003	0.01	Oxamyl	0.003	0.01	Tolclofos-methyl	0.003	0.01
Chrysene	0.003	0.01	Fenoxycarb	0.003	0.01	Oxidemeton-methyl	0.003	0.01	Tolylfluanid	0.003	0.01
Clofentezine	0.003	0.01	Fenpropathrin	0.003	0.01	Oxyfluorfen	0.003	0.01	Tralomethrin	0.003	0.01
Clomazone	0.003	0.01	Fenpropidin	0.003	0.01	Paclobutrazol	0.003	0.01	Transfluthrin	0.003	0.01
Clorthal-dimethyl	0.003	0.01	Fenpropimorph	0.003	0.01	Paraffin chlorinated	0.003	0.01	Triadimefon	0.003	0.01
Cyanazine	0.003	0.01	Fensulfothion	0.003	0.02	Parathion ethyl	0.003	0.01	Triadimenol	0.003	0.01
Cyfluthrin	0.003	0.01	Fenthion	0.003	0.01	Parathion methyl	0.003	0.01	Tri-allate	0.003	0.01
Cyfluthrin-beta	0.003	0.01	Fenthion	0.003	0.01	PCB 180	0.003	0.01	Triazophos	0.003	0.01
Cyhalothrin Gamma	0.003	0.01	Fenvalerate	0.003	0.01	Penconazole	0.003	0.01	Tribromophenol	0.003	0.01
Cyhalothrin lambda	0.003	0.01	Fipronil	0.003	0.01	Pendimethalin	0.003	0.01	Trichlorfon	0.003	0.01
Cypermethrin	0.003	0.01	Fluazifop –P-butyl	0.003	0.01	Permethrin	0.003	0.01	Tricresyl phosphate	0.003	0.01
Cyproconazole	0.003	0.01	Fluazinam	0.003	0.01	Phenmedipham	0.006	0.02	Tridemorph	0.003	0.01
Cyprodinil	0.003	0.01	Flucythrinate	0.003	0.01	Phenothrin	0.003	0.01	Trifloxystrobin	0.003	0.01
Cyromazine	0.003	0.01	Fludioxonil	0.003	0.01	Phorate	0.003	0.01	Triflumizole	0.003	0.01
Dalapon-sodio	0.003	0.01	Flufenoxuron	0.003	0.01	Phosalone	0.003	0.01	Trifluralin	0.003	0.01
DDD-o,p’	0.003	0.01	Fluquinconazole	0.003	0.01	Phosmet	0.003	0.01	Vamidothion	0.003	0.01
DDD-p,p’	0.003	0.01	Flurochloridone	0.003	0.01	Phosphamidon	0.003	0.01	Vinclozolin	0.003	0.01
DDE-o,p’	0.003	0.01	Fluroxypyr 1-methylheptyl ester	0.003	0.01	Picloram	0.003	0.01			
